# Supplementary figures and images for: Mendelian Randomization Studies: A Metric for Quality Evaluation
Source: Gout Urate Cryst Depos Dis. Author manuscript; Available in PMC 2026 Jul 23. (PMC13390894; doi:10.3390/gucdd3020008)

## Supplement. Quick Guide: Is This Mendelian Randomization Study Reliable?

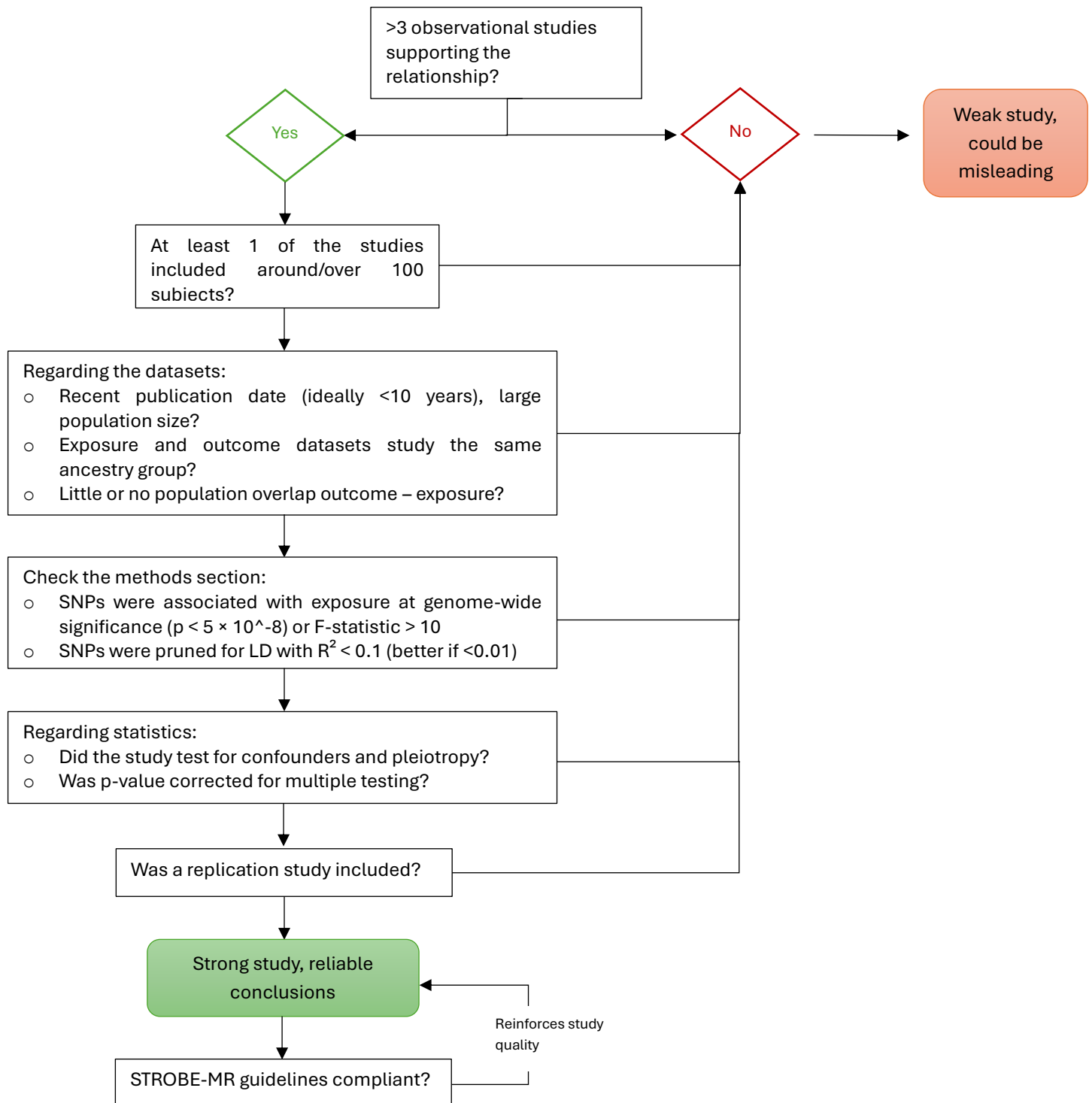

Supplement: Supplemental Figure 1 [file NIHMS2193350-supplement-Supplemental_Figure_1.pdf]
